# Supplementary material for: Benchmarking Farm Animal Welfare—A Novel Tool for Cross-Country Comparison Applied to Pig Production and Pork Consumption
Source: Animals (Basel). 2020 May 31;10(6):955. doi: 10.3390/ani10060955 (PMC7341196; doi:10.3390/ani10060955)
Supplement: Supplementary file 1 [file animals-10-00955-s001.zip › Table S2- References to webpages about pig welfare initiatives.pdf]

Table S4: References to webpages about pig welfare initiatives

SE
http://www.jordbruksverket.se
https://www.coop.se
https://www.svensktsigill.se/

DE
http://www.neuland-fleisch.de
https://initiative-tierwohl.de
https://www.haltungsform.de/

UK
https://www.gov.uk/government/statistics/organic-farming-statistics-2018
https://www.greenpasturefarms.co.uk
https://science.rspca.org.uk
https://www.soilassociation.org
https://www.compassioninfoodbusiness.com/awards/good-pig-award/
https://assurance.redtractor.org.uk
https://www.qmscotland.co.uk
http://www.legislation.gov.uk/uksi/2007/2078/schedule/8/made

NL
https://www.nvwa.nl/
https://www.boerenbusiness.nl
https://www.hamletz.nl/het-merk/
https://www.frievar.com
https://www.livar.nl
http://degroeneweg.nl

DK
https://www.foedevarestyrelsen.dk
https://lbst.dk
https://www.friland.dk
https://www.lf.dk
http://hestbjerg.dk/
