# Supplementary material for: Benchmarking Farm Animal Welfare—A Novel Tool for Cross-Country Comparison Applied to Pig Production and Pork Consumption
Source: Animals (Basel). 2020 May 31;10(6):955. doi: 10.3390/ani10060955 (PMC7341196; doi:10.3390/ani10060955)
Supplement: Supplementary file 1 [file animals-10-00955-s001.zip › Table S1- Key figures for the pig industries.pdf]

Table S7: Key figures for the pig industries

The pig industries are significantly different in the countries included in this study, and this can be factors explaining a part of the benchmark values. The size of the industry, the international trade and the farm structure for all five countries are shown below. When benchmark value for domestic consumption is calculated, domestic consumption is estimated as production + import – export -/+ stock changes. "Domestic supply" as shown in the table is used as an approximation of consumption.

Table: Key figures for the pig industry in the countries included in this study

	1)	1)	1)	1)	1)	1)	2)
					Pig meat	Self	
	Pigs	Pig meat	Pig meat	Pig meat	Domestic	sufficiency	Pigs per
	stock	Production	Export	Import	supply	rate	pig holding
	Million	Million tons	1.000 tons	1.000 tons	1.000 tons	Percent	Pigs
Year	2018	2018	2017	2017	2017	2017	2013
**DK**	13	2	1.306	126	240	660	3.128
**D**	26	5	2.355	1.121	4.170	129	584
**NL**	12	1	1.164	371	636	230	2.208
**ES**	31	5	1.827	185	2.501	181	467
**S**	1	0	30	120	330	75	1.093
**UK**	5	1	262	1.007	1.649	56	473
**EU**	150	24	10.273	7.641	21.068	114	66

1) Source: FAOSTAT (2020)

2) Source: Eurostat (2020)
