# Supplementary material for: Benchmarking Farm Animal Welfare—A Novel Tool for Cross-Country Comparison Applied to Pig Production and Pork Consumption
Source: Animals (Basel). 2020 May 31;10(6):955. doi: 10.3390/ani10060955 (PMC7341196; doi:10.3390/ani10060955)
Supplement: Supplementary file 1 [file animals-10-00955-s001.zip › Table S6- Calculation of coefficients.pdf]

Table S5: Calculation of coefficients

The stock of pigs can be divided into piglets, fattening pigs, sows, etc. in different age groups. Animal welfare characteristics relate to different parts of the production or herd. Some countries have a relatively large piglet production, so in these countries the animal welfare characteristics found in piglet production should be given greater weight. The relative size of the different groups of pigs is calculated, and weights are estimated. Data input and calculations are presented below.

Table: Composition of pig population

**Population, 1.000 animals**						
	Denmark	Germany	Netherlands	Sweden	United Kingdom	Total
Live swine, domestic species	12.642	26.445	11.934	1.417	4.648	57.087
Piglets, less than 20 kg	4.543	7.672	5.307	355	1.216	19.093
Breeding pigs	1.254	1.855	978	125	502	4.714
Breeding sows	1.243	1.837	970	123	490	4.663
Covered sows	775	1.314	642	81	340	3.153
Sows covered for the first time	190	209	108	58	55	620
Sows, not covered	468	523	328	42	150	1.510
Gilts not yet covered	223	226	134	23	84	690
Pigs, from 20 kg to less than 50 kg	3.937	5.049	1.617	369	1.187	12.159
Fattening pigs, 50 kg or over	2.908	11.870	4.033	568	1.743	21.123
Fattening pigs, from 50 kg to less than 80 kg	2.321	5.360	1.666	239	1.023	10.609
Fattening pigs, from 80 kg to less than 110 kg	580	5.284	1.679	231	664	8.438
Fattening pigs, 110 kg or over	7	1.227	687	98	56	2.075
Breeding boars	11	18	8	1	12	50
**Population, 1.000 animals**						
	Denmark	Germany	Netherlands	Sweden	United Kingdom	
Sows	4.153	5.964	3.160	452	1.621	15.349
Piglets	8.480	12.720	6.924	724	2.403	31.252
Fattening pigs	5.816	23.741	8.065	1.137	3.486	42.244
**Population, percent**						
	Denmark	Germany	Netherlands	Sweden	United Kingdom	Average
Sows	22,5	14,1	17,4	19,5	21,6	17,3
Piglets	46,0	30,0	38,2	31,3	32,0	35,2
Fattening pigs	31,5	56,0	44,4	49,1	46,4	47,5
**Population, coefficient**						
	Denmark	Germany	Netherlands	Sweden	United Kingdom	Average
Sows	1,3	0,8	1,0	1,1	1,2	1,0
Piglets	1,3	0,9	1,1	0,9	0,9	1,0
Fattening pigs	0,7	1,2	0,9	1,0	1,0	1,0
All	1,0	1,0	1,0	1,0	1,0	1,0

Source: Eurostat (2020)
