# Supplementary material for: Benchmarking Farm Animal Welfare—A Novel Tool for Cross-Country Comparison Applied to Pig Production and Pork Consumption
Source: Animals (Basel). 2020 May 31;10(6):955. doi: 10.3390/ani10060955 (PMC7341196; doi:10.3390/ani10060955)
Supplement: Supplementary file 1 [file animals-10-00955-s001.zip › Table S7- Import composition of domestic consumption assessments.pdf]

Table S6: Import composition of domestic consumption assessments

As import of pigmeat is significant, both import volume, import countries and import as a share of domestic consumption is important. These figures (plus production, export etc.) are shown in this file.

Assumptions regarding import shares, consumption etc.

Sweden

**Supply balance**	1.000 tons
Production	241
Import Quantity	117
Export Quantity	29
Domestic supply quantity	330
Import - percent of production	35
Import - percent of consumption	49

**Import from major countries (USD, 2017)**		
Germany	112.846.386	38,0
Denmark	82.571.807	27,8
Poland	48.816.630	16,4
Netherlands	27.298.928	9,2
UK	3.849.814	1,3
Spain	3.331.035	1,1
Other	18.548.383	6,2
Total	297.262.983	100,0

Germany

**Supply balance**	1.000 tons
Production	5506
Import Quantity	1191
Export Quantity	2563
Domestic supply quantity	4170
Import - percent of production	29
Import - percent of consumption	22

**Import from major countries (USD, 2017)**		
Belgium	593.927.161	32,2
Denmark	538.714.377	29,2
Netherlands	296.344.578	16,1
Spain	137.012.476	7,4
Poland	101.953.315	5,5
UK	47.253.394	2,6
France	38.895.465	2,1
Other	91.873.894	5,0
Total	1.845.974.660	100,0

Netherlands

**Supply balance**	1.000 tons
Production	1456
Import Quantity	392
Export Quantity	1201
Domestic supply quantity	636
Import - percent of production	62
Import - percent of consumption	27

**Import from major countries (USD, 2017)**		
Germany	465.943.748	66,2
Belgium	83.421.574	11,9
France	38.174.192	5,4
Poland	34.953.601	5,0
Denmark	20.006.976	2,8
Other	60.882.035	8,7
Total	703.382.126	100,0

Denmark

**Supply balance**	1.000 tons
Production	1532
Import Quantity	137
Export Quantity	1397
Domestic supply quantity	240
Import - percent of production	57
Import - percent of consumption	9

**Import from major countries (USD, 2017)**		
Germany	101.967.911	70,1
Netherlands	10.749.200	7,4
Spain	9.472.531	6,5
Norway	5.600.672	3,8
Spain	5.242.626	3,6
Other	12.528.624	8,6
Total	145.561.564	100,0

U.K.

**Supply balance**	1.000 tons
Production	901
Import Quantity	979
Export Quantity	262
Domestic supply quantity	1649
Import - percent of production	59
Import - percent of consumption	109

**Import from major countries (USD, 2017)**		
Denmark	385.895.356	32,0
Germany	254.218.324	21,1
Netherlands	130.893.527	10,8
Spain	115.900.769	9,6
Ireland	104.956.242	8,7
Belgium	80.371.213	6,7
France	59.824.572	5,0
Other	75.192.023	6,2
Total	1.207.252.026	100,0
