# Supplementary material for: Benchmarking Farm Animal Welfare—A Novel Tool for Cross-Country Comparison Applied to Pig Production and Pork Consumption
Source: Animals (Basel). 2020 May 31;10(6):955. doi: 10.3390/ani10060955 (PMC7341196; doi:10.3390/ani10060955)
Supplement: Supplementary file 1 [file animals-10-00955-s001.zip › Table S5- Benchmark matrix combining grades of animal welfare and expert assessments.pdf]

Table S4: Benchmark matrix combining grades of animal welfare and expert assessments

A key element in the benchmark value estimation is the matrix that combines grades of animal welfare of the 15 dimensions, expert assessment of each grade and assessment of the importance of each dimension of animal welfare. First step in the calculation is to estimate a benchmark value for each single initiative (legislation or label) in each country. Such a benchmark value for each single initiative in each country is calculated from following input:

1) Label characteristics (X): For each initiative, a grade is assigned for each welfare dimension. As the same grades are used to categorize all initiatives, some of the assigned grades are not precise but the closest possible that fit the existing labels.
2) Expert assessments of grades (V): The animal welfare score for each grade within a dimension. Values from 0 to 10.
3) Expert assessments of importance of dimensions (I): Values 1 to 5.
4) Coefficients (Z): Animal welfare characteristics relate to different parts of the production or herd. In the pig sector, for example, the piglet, finishers and sow population are included. Some countries have a relatively large piglet production, so in these countries the animal welfare characteristics found in piglet production should be given greater weight. These weights are denoted coefficients in the table.

The benchmark value of the individual initiative (legislation or label) in each country is obtained by multiplying the four mentioned factors for dimension and aggregating over all dimensions. A low value of a welfare dimension (close to zero) is obtained if the animal welfare characteristics found are low in the groupings and / or if the groupings concerned have a low weight.

Calculation of benchmark value are made in a matrix in spreadsheets. A generic example is shown in Table S4.

Table S4. Spreadsheet showing input for calculating benchmark value for individual labels

								Country A					Country B				. . . .	Country N			
		Expert No.1	Expert No. 2	Expert . . .	Expert No. n	Expert Average		Label No. 1	Label No. 2	Label . .	Label No. N		Label No. 1	Label No. 2	Label . .	Label No. N		Label No. 1	Label No. 2	Label . .	Label No. N
								**A1**	**A2**		**An**		**B1**	**B2**		**Bn**		**N1**	**N2**		**Nn**
**1. Dimension**																					
	No regulation					V1-1															
	Minimun EU regulation					V1-2		X										X			
	Low level					V1-3			X				X	X							
	.																		X		
	.															X					X
	Highest level					V1-n					X										
	Importance					I-1															
**2. Dimension**																					
	No regulation					V2-1															
	Minimun EU regulation					V2-2		X	X				X								
	Low level					V2-3												X			
	.													X					X		
	.										X					X					
	Highest level					V2-n															X
	Importance					I-2															
**n. Dimension**																					
	No regulation					Vn-1															
	Minimun EU regulation					Vn-2		X	X				X					X			
	Low level					Vn-3															
	.													X							
	.										X								X		
	Highest level					Vn-n										X					X
	Importance					I-m															

X illustrate which grades in each dimension that characterize each label. V and I are weights provided by expert. A_1 . . .A_n, B_1 . . . B_n and N_1 . . . N_n are benchmark values for individual labels in countries A, B and N.

The benchmark value of the total production of a given product in a country is calculated by weighing the market shares of benchmark value of the individual labels. The market shares may be the production and consumption market shares. The estimation of benchmark value for one country includes more than 220 different numbers from different sources. The files are linked and are connected automatically.

Please, find the entire matrix with grades, expert assessments, weights etc. on next page.

Important: Enlarge the text to at least 400 percent in order to read the matrix.
