# Supplementary material for: Benchmarking Farm Animal Welfare—A Novel Tool for Cross-Country Comparison Applied to Pig Production and Pork Consumption
Source: Animals (Basel). 2020 May 31;10(6):955. doi: 10.3390/ani10060955 (PMC7341196; doi:10.3390/ani10060955)
Supplement: Supplementary file 1 [file animals-10-00955-s001.zip › Table S3- Overview of labels etc-final.pdf]

Table S2: Overview of animal welfare labels, market shares and import export for The Netherlands, Sweden, Germany, United Kingdom and Denmark

The Netherlands

A few introductory comments. The market shares of some of the labels are not represented individually in the calculation of market shares of welfare labels.

Dutch law (IKB Varken and Heyde Hoeve are categorized here as having the same animal welfare as Dutch law). IKB Varken covers almost is all production (95%). Mainly food safety and biosecurity.
BL1 (Keten Duurzam Varkenvlees KDV (Sustainable Pork Value Chain), Good farming star, Good farming welfare, Frievar-Friberne, Scharrelvarken, Livar, Wroetvarken are categorized here as having the same animal welfare as BL1). Note KDV has 2 levels, KDV1 does not qualify for BL1 – only KDV2 does but they are both categorized here as BL1, but the volume is small.
BL2 (Hamletz is categorized here as having the same animal welfare as BL2)
BL3 (Livar and De groene Weg are categorized here as having the same animal welfare as BL3)
Organic (is perceived here to provide the same animal welfare as BL3 but is represented separately)

Table S2.1 Overview of market shares for animal welfare labelled pork in the Netherlands

	Introduced	Market shares of consumption (volume)	Share of production (volume)	Institution
**Dutch law**		0%	5%	
**IKB Varken**	1995	22% of consumption (=100-75-3)	73% of production (=95-20-2)	
**Beter Leven (Better Life)**<br>BL1 indoor<br>BL2 Free range<br>BL3 Organic	2008 (pigs)	75% of pork consumption in 2019 hereof<br>BL1– 97% of BL<br>BL2 0,1% of BL<br>BL3 3% of BL	20% of production (3.6 mio. Pigs) in 2019<br>BL1 99% of BL<br>BL2 0% of BL<br>BL3 1% of BL	Dutch Society for protection of animals
**Keten duurzam Varkenvlees KDV**<br>KDV1 below BL1<br>KDV2 as BL1	1997	Share of consumption – reported under BL1	(2.7% of production)<br>Reported under BL1	KDV
**Good farming star**<br>as BL1	2010	Share of consumption – reported under BL1	(5.3% of production)<br>Reported under BL1	VION
**Good farming welfare**<br>as BL1	Ca. 2010	0%	1 million finishers in 2014<br>Reported under BL1	VION (only export to the British market)
**Wroetvarken**<br>as BL1	2019	Share of consumption – reported under BL1	40.000 pigs<br>Reported under BL1	
**Hamletz**<br>as BL2		Share of consumption – reported under BL2	5000 pigs<br>Reported under BL2	
**Frievar-Friberne**	2002	Not included	50.000 pigs<br>Not included	WelfareQuality assessment
**Livar**<br>As BL3	1999	Share of consumption reported under BL3	7.000 pigs<br>Reported under BL3	Livar
**Heide Hoeve**<br>Below BL1		Reported under IKB Varken	30.000 pigs<br>Reported under IKB	
**Organic (EKO)**<br>as BL 3		3% of consumption	2%	SKAL (De Groene weg)
**Import and export**		22% of consumption	45% of production	

Note:
The Netherlands import of pork amounts to approx. 50 per cent. of consumption. As the case with Denmark, a significant part is probably re-exported. We have assumed that 22 per cent of consumption is the import of foreign-produced pork for human consumption in the Netherlands. Thereby, IKB Varken market shares can be calculated as the residual (22=100-75-3).

Sweden

Table S2.2 Table 1 Overview of market shares for animal welfare labelled pork in Sweden

	Introduced	Market shares of consumption (volume)	Share of production (volume)	Institution
**Swedish law**		62% of consumption (= 100-35-1,5-0,75- 0,75)	97% of production (=100-3)	Public authorities
**IP Sigill**	1992	0,75% of consumption	1% of production	Private
**John's Selection**	2017	0,75% of consumption	0% of production	Sold in COOP (Dutch origin)
**Organic**	1988	1,5 % of consumption	2% of production	KRAV (private)
**Import and export**		35% of consumption	12% of production	

Germany

A few introductory comments

QS is not included as the welfare criteria are almost identical to German national law. 95% of the German pig production is QS certified

Initiative Tierwohl is included as follows. There are two levels of IT. We assume that 50% comply with the voluntary criteria IT2 and 50% comply with base level IT1.

Organic There are a number of different organic labels in Germany that all guarantee at least that the EU minimum requirements are satisfied (Naturland, Demeter, Bioland and maybe more). All are gathered in "EU organic".

Tierschutz Kontrolliert from the Vier Pfoten organization is not yet marketed for pigs and is therefore not included.

Tierwohl kennzeichen The coming state label is not included as it is not yet marketed.

Haltungsform serves to classify existing labels into four categories: H1 equivalent to QS, H2 as IT1, H3 as T1, H4 as T2. Haltungsform is not registered as a separate label.

Table S2.3 Overview of market shares for animal welfare labelled pork in Germany

	**Introduced**	**Market shares of consumption**	**Market share of production**	**Institution**
**German law**		**0%**	**5% of production**	
**QS**		**46% of consumption**<br>(=100-27-0.2-0.1-1-25)	**74% of production**<br>(=95-20-0.1-0.05-0.6)	
**Initiative Tierwohl**<br>IT1 base<br>IT2 voluntary	2017	IT1 12.5 %<br>IT2 12.5 %	IT1 10 %<br>IT2 10 %	Private initiative
**Für mehr Tierschutz**<br>T1 indoor<br>T2 outdoor	2013<br>Only for finishers	T1 0.1 %<br>T2 0.1 %	T1 0.05 %<br>T2 0.05 %	Deutches Tierschutzbund
**Haltungsform**<br>H1 as QS<br>H2 as IT1<br>H3 as T1<br>H4 as T2	April 2018: Lidl Haltungskompass.<br>April 2019: Haltungsform more super markets	**In total 25% but registered under the respective labels**<br>H1 11 %<br>H2 11 %<br>H3 1.5 %<br>H4 1.5 %	**In total 20% but registered under the respective labels**<br>H1 9 %<br>H2 9 %<br>H3 1 %<br>H4 1 %	Retail
**Neuland**<br>outdoor	1989	**0.1%**	**0.05%**	Private, Deutches Tierschutzbund
**Organic**		**1%**	**0.6%**	Various private labels
**Import and export**		27% of consumption imported	44% of production exported	

Table S2.4 Overview of market shares for animal welfare labelled pork in the UK

	**Introduced**	**Market shares of consumption**	**Share of production**	**Institution**
UK common law		0%	5%	
Red tractor		12%=100-62-20-5-1	44% (=95-40-10-1)	Private
RSPCA assured<br>R1 indoor<br>R2 outdoor bred<br>R3 outdoor reared<br>R4 free-range finished	1994 Freedom Food (name change to RSPCA assured in 2016)	**R1 5%**<br>**R2-R4 20% of consumption outdoors divided into**<br>R2 14% outdoor bred<br>R3 5% outdoor reared<br>R4 1% free-range finished	**R1 10%**<br>**R2-R4 40% of production outdoors divided into**<br>R2 27% outdoor bred<br>R3 10% outdoor reared<br>R4 3% free-range finished	Private
Organic	1946	1%	1%	Soil association, Organic farmers and Growers
Import and export (2018 figures)		62% of consumption is imported	29% of production is exported	

Note: The countries that the UK import from and import volumes are shown in supplementary material S2.

Table S2.5 Overview of market shares for animal welfare labelled pork in Denmark

	**Introduced**	**Market shares of consumption**	**Share of production**	**Institution**
**Danish law**		0%	5%	
**Danish brand**		70% of consumption (=100-10-20)	88% of production (=100-6-0,01-0,3-0,7)	
**Better Animal welfare**<br>F1 indoor<br>F2 indoor<br>F3 outdoor	2017	7% of consumption<br>F1 0%<br>F2 5%<br>F3 2%	0,7% of production<br>F1 0%<br>F2 0,5%<br>F3 0,2%	Public authorities Danish Veterinary and Food Administration (DVFA)
**Welfare heart**<br>C1 indoor<br>C2 outdoor<br>C3 outdoor as organic<br>C4 outdoor extra	2016	3% of consumption<br>C1 0,9%<br>C2 0,9%<br>C3 2%<br>C4 0,2%	0,3% of production<br>C1 0,1%<br>C2 0,1%<br>C3-C4 0,1%	Private (COOP)
**Danish organic**		0,1%	0,01%	Public authorities
**UK pigs**		0%	6% of production	Private
**Import and export**		20% of consumption is imported	90% of production is exported	

Notes: Altogether approx. 10% of consumption is welfare pork hereof 6% indoor welfare and 4% outdoor welfare. Denmark's import of pork amounts to approx. 50 per cent. of consumption. However, a significant part is re-import of pork processing abroad or it is re-exported. It is therefore assumed that 20 per cent of consumption is the import of foreign-produced pork for human consumption in Denmark

Table S2.6 Import share of consumption and export share of production in each country

	Import share of consumption	Export share of production
DK	53%	83%
D	27%	44%
NL	58%	80%
ES	7%	40%
S	36%	12%
UK	61%	28%
EU	36%	43%

Note: Estimated based on key figures for pig production from FAOSTAT (2020) – the data are from 2017 or 2018
