# Supplementary material for: Benchmarking Farm Animal Welfare—A Novel Tool for Cross-Country Comparison Applied to Pig Production and Pork Consumption
Source: Animals (Basel). 2020 May 31;10(6):955. doi: 10.3390/ani10060955 (PMC7341196; doi:10.3390/ani10060955)
Supplement: Supplementary file 1 [file animals-10-00955-s001.zip › Table S4 - Expert questionnaire final.pdf]

Table S3: Expert questionnaire on animal welfare in pig production

Thank you for agreeing to participate in our survey.

We would like you to evaluate the animal welfare effects of various initiatives for pig production in comparison to the EU regulations. The questionnaire is divided into four themes:

Welfare for the sow
Welfare for the piglet
Welfare for weaners and finishers
Welfare issues that span different categories

For each question below, we ask you to assign a number between 0 and 10, where:

0 represents minimal welfare for the pigs
10 represents the best possible welfare for the pigs

The assumption in every question is that all other aspects are equal, so that you only evaluate one aspect of the production systems in each question.

In the last part of the questionnaire, we ask you to evaluate how important the individual aspects of the production systems are for pig welfare as a whole.

Informed consent

By taking part in this survey you consent that the data from this survey will be used in our studies, and you understand that all analysis and presentation of data from this survey will be anonymous and in no way tracked back to you personally. Do you consent with these conditions?

(1) ❑ I consent
(2) ❑ I do not consent

SOWS

What score would you give for <u>sow welfare</u> in a production system where the sow is confined for certain periods? The scale is from 0 to 10 where 0 represents minimal welfare for the sow and 10 represents the best possible welfare for the sow regarding this feature.

Note: The welfare of the piglets in this production system is addressed in another question.

	**0**	**1**	**2**	**3**	**4**	**5**	**6**	**7**	**8**	**9**	**10**	**Do not know**
The sow is confined at all times (does not meet EU requirements)	(2) ❑	(3) ❑	(4) ❑	(12) ❑	(13) ❑	(14) ❑	(15) ❑	(16) ❑	(17) ❑	(18) ❑	(19) ❑	(20) ❑
The sow is loose in the gestation unit (i.e. loose 60% of the time) but confined in the mating and farrowing units (meets EU requirements)	(2) ❑	(3) ❑	(4) ❑	(12) ❑	(13) ❑	(14) ❑	(15) ❑	(16) ❑	(17) ❑	(18) ❑	(19) ❑	(20) ❑
The sow is loose in the mating and gestation units (i.e. loose 80% of the time) but confined in the farrowing unit	(2) ❑	(3) ❑	(4) ❑	(12) ❑	(13) ❑	(14) ❑	(15) ❑	(16) ❑	(17) ❑	(18) ❑	(19) ❑	(20) ❑
The sow is loose at all times except 4-5 days around farrowing	(2) ❑	(3) ❑	(4) ❑	(12) ❑	(13) ❑	(14) ❑	(15) ❑	(16) ❑	(17) ❑	(18) ❑	(19) ❑	(20) ❑
The sow is loose at all times except 2-3 day around farrowing	(2) ❑	(3) ❑	(4) ❑	(12) ❑	(13) ❑	(14) ❑	(15) ❑	(16) ❑	(17) ❑	(18) ❑	(19) ❑	(20) ❑
The sow is loose at all times	(2) ❑	(3) ❑	(4) ❑	(12) ❑	(13) ❑	(14) ❑	(15) ❑	(16) ❑	(17) ❑	(18) ❑	(19) ❑	(20) ❑
The sow is loose at all times and she farrows in huts with outdoor access	(2) ❑	(3) ❑	(4) ❑	(12) ❑	(13) ❑	(14) ❑	(15) ❑	(16) ❑	(17) ❑	(18) ❑	(19) ❑	(20) ❑

What score would you give for sow welfare in a production system with the following space requirements for sows with piglets? The scale is from 0 to 10 where 0 represents minimal welfare for the sow and 10 represents the best possible welfare for the sow.

Note: The welfare of the piglets in this production system is addressed in another question.

	**0**	**1**	**2**	**3**	**4**	**5**	**6**	**7**	**8**	**9**	**10**	**Do not know**
3 m2 per sow (below the EU norm)	(2) ❑	(3) ❑	(4) ❑	(12) ❑	(13) ❑	(14) ❑	(15) ❑	(16) ❑	(17) ❑	(18) ❑	(19) ❑	(20) ❑
4 m2 per sow (EU norm)	(2) ❑	(3) ❑	(4) ❑	(12) ❑	(13) ❑	(14) ❑	(15) ❑	(16) ❑	(17) ❑	(18) ❑	(19) ❑	(20) ❑
6 m2 per soW	(2) ❑	(3) ❑	(4) ❑	(12) ❑	(13) ❑	(14) ❑	(15) ❑	(16) ❑	(17) ❑	(18) ❑	(19) ❑	(20) ❑
7.5 m2 per sow	(2) ❑	(3) ❑	(4) ❑	(12) ❑	(13) ❑	(14) ❑	(15) ❑	(16) ❑	(17) ❑	(18) ❑	(19) ❑	(20) ❑
10 m2 per sow	(2) ❑	(3) ❑	(4) ❑	(12) ❑	(13) ❑	(14) ❑	(15) ❑	(16) ❑	(17) ❑	(18) ❑	(19) ❑	(20) ❑
10 m2 per sow, with outdoor access	(2) ❑	(3) ❑	(4) ❑	(12) ❑	(13) ❑	(14) ❑	(15) ❑	(16) ❑	(17) ❑	(18) ❑	(19) ❑	(20) ❑

What score would you give for sow welfare in a production system with the following space requirements for sows in group housing? The scale is from 0 to 10 where 0 represents minimal welfare for the sow and 10 represents the best possible welfare for the sow.

	**0**	**1**	**2**	**3**	**4**	**5**	**6**	**7**	**8**	**9**	**10**	**Do not know**
1.5 m2 per sow (does not meet EU requirements)	(2) ❑	(3) ❑	(4) ❑	(12) ❑	(13) ❑	(14) ❑	(15) ❑	(16) ❑	(17) ❑	(18) ❑	(19) ❑	(20) ❑
2.25 m2 per sow (meets EU requirements for groups under 40 sows; the EU	(2) ❑	(3) ❑	(4) ❑	(12) ❑	(13) ❑	(14) ❑	(15) ❑	(16) ❑	(17) ❑	(18) ❑	(19) ❑	(20) ❑

	0	1	2	3	4	5	6	7	8	9	10	Do not know
standard for groups of over 40 sows is 2.025 m2)												
2.5 m2 per sow	(2) ❑	(3) ❑	(4) ❑	(12) ❑	(13) ❑	(14) ❑	(15) ❑	(16) ❑	(17) ❑	(18) ❑	(19) ❑	(20) ❑
2.8 m2 per sow	(2) ❑	(3) ❑	(4) ❑	(12) ❑	(13) ❑	(14) ❑	(15) ❑	(16) ❑	(17) ❑	(18) ❑	(19) ❑	(20) ❑
3 m2 per sow	(2) ❑	(3) ❑	(4) ❑	(12) ❑	(13) ❑	(14) ❑	(15) ❑	(16) ❑	(17) ❑	(18) ❑	(19) ❑	(20) ❑
3 m2 per sow, with outdoor access	(2) ❑	(3) ❑	(4) ❑	(12) ❑	(13) ❑	(14) ❑	(15) ❑	(16) ❑	(17) ❑	(18) ❑	(19) ❑	(20) ❑

What score would you give for sow welfare in a production system with the following access to nest-building material? The scale is from 0 to 10 where 0 represents minimal welfare for the sow and 10 represents the best possible welfare for the sow.

	0	1	2	3	4	5	6	7	8	9	10	Do not know
Sows are not given any nest-building material (does not meet EU requirements)	(2) ❑	(3) ❑	(4) ❑	(12) ❑	(13) ❑	(14) ❑	(15) ❑	(16) ❑	(17) ❑	(18) ❑	(19) ❑	(20) ❑
The week before farrowing, sows are provided with appropriate nest-building material unless it is technically not possible due to the slurry system (meets EU requirements)	(2) ❑	(3) ❑	(4) ❑	(12) ❑	(13) ❑	(14) ❑	(15) ❑	(16) ❑	(17) ❑	(18) ❑	(19) ❑	(20) ❑
The week before farrowing, sows are provided with appropriate nest-building material (no dispensations)	(2) ❑	(3) ❑	(4) ❑	(12) ❑	(13) ❑	(14) ❑	(15) ❑	(16) ❑	(17) ❑	(18) ❑	(19) ❑	(20) ❑

	0	1	2	3	4	5	6	7	8	9	10	Do not know
The week before farrowing, sows are provided with plenty of nest-building material (no dispensations)	(2) ❑	(3) ❑	(4) ❑	(12) ❑	(13) ❑	(14) ❑	(15) ❑	(16) ❑	(17) ❑	(18) ❑	(19) ❑	(20) ❑

What score would you give for <u>sow welfare</u> in a production system with the following practice for weaning? The scale is from 0 to 10 where 0 represents minimal welfare for the sow and 10 represents the best possible welfare for the sow.

Note: The welfare of the piglets in this production system is addressed in another question.

	0	1	2	3	4	5	6	7	8	9	10	Do not know
Piglets are weaned after 14 days (does not meet EU requirements)	(2) ❑	(3) ❑	(4) ❑	(12) ❑	(13) ❑	(14) ❑	(15) ❑	(16) ❑	(17) ❑	(18) ❑	(19) ❑	(20) ❑
Piglets are weaned after 21 days (EU requirement is 28 days but special dispensation to 21 days can be given)	(2) ❑	(3) ❑	(4) ❑	(12) ❑	(13) ❑	(14) ❑	(15) ❑	(16) ❑	(17) ❑	(18) ❑	(19) ❑	(20) ❑
Piglets are weaned after 28 days	(2) ❑	(3) ❑	(4) ❑	(12) ❑	(13) ❑	(14) ❑	(15) ❑	(16) ❑	(17) ❑	(18) ❑	(19) ❑	(20) ❑
Piglets are weaned after 30 days	(2) ❑	(3) ❑	(4) ❑	(12) ❑	(13) ❑	(14) ❑	(15) ❑	(16) ❑	(17) ❑	(18) ❑	(19) ❑	(20) ❑
Piglets are weaned after 40 days	(2) ❑	(3) ❑	(4) ❑	(12) ❑	(13) ❑	(14) ❑	(15) ❑	(16) ❑	(17) ❑	(18) ❑	(19) ❑	(20) ❑
Piglets are weaned after 49 days	(2) ❑	(3) ❑	(4) ❑	(12) ❑	(13) ❑	(14) ❑	(15) ❑	(16) ❑	(17) ❑	(18) ❑	(19) ❑	(20) ❑
Piglets are weaned after 70 days	(2) ❑	(3) ❑	(4) ❑	(12) ❑	(13) ❑	(14) ❑	(15) ❑	(16) ❑	(17) ❑	(18) ❑	(19) ❑	(20) ❑

PIGLETS

What score would you give for <u>piglet welfare</u> in a production system where the sow is confined for certain periods with different space requirements? The scale is from 0 to 10 where 0 represents minimal welfare for the piglet and 10 represents the best possible welfare for the piglet regarding this feature.
Note: The welfare of the sow in this production system is addressed in another question.

Piglet welfare when the sow is confined in the farrowing pen:

	**0**	**1**	**2**	**3**	**4**	**5**	**6**	**7**	**8**	**9**	**10**	**Do not know**
With pens of 4 m2 (as per EU norm)	(2) ❑	(3) ❑	(4) ❑	(12) ❑	(13) ❑	(14) ❑	(15) ❑	(16) ❑	(17) ❑	(18) ❑	(19) ❑	(20) ❑
With pens of 6 m2	(2) ❑	(3) ❑	(4) ❑	(12) ❑	(13) ❑	(14) ❑	(15) ❑	(16) ❑	(17) ❑	(18) ❑	(19) ❑	(20) ❑
With pens of 7.5 m2	(2) ❑	(3) ❑	(4) ❑	(12) ❑	(13) ❑	(14) ❑	(15) ❑	(16) ❑	(17) ❑	(18) ❑	(19) ❑	(20) ❑
With pens of 10 m2	(2) ❑	(3) ❑	(4) ❑	(12) ❑	(13) ❑	(14) ❑	(15) ❑	(16) ❑	(17) ❑	(18) ❑	(19) ❑	(20) ❑

Piglet welfare when the sow is loose in the farrowing pen, except for 4-5 days after farrowing:

	**0**	**1**	**2**	**3**	**4**	**5**	**6**	**7**	**8**	**9**	**10**	**Do not know**
With pens of 4 m2 (as per EU norm)	(2) ❑	(3) ❑	(4) ❑	(12) ❑	(13) ❑	(14) ❑	(15) ❑	(16) ❑	(17) ❑	(18) ❑	(19) ❑	(20) ❑
With pens of 6 m2	(2) ❑	(3) ❑	(4) ❑	(12) ❑	(13) ❑	(14) ❑	(15) ❑	(16) ❑	(17) ❑	(18) ❑	(19) ❑	(20) ❑
With pens of 7.5 m2	(2) ❑	(3) ❑	(4) ❑	(12) ❑	(13) ❑	(14) ❑	(15) ❑	(16) ❑	(17) ❑	(18) ❑	(19) ❑	(20) ❑
With pens of 10 m2	(2) ❑	(3) ❑	(4) ❑	(12) ❑	(13) ❑	(14) ❑	(15) ❑	(16) ❑	(17) ❑	(18) ❑	(19) ❑	(20) ❑

Piglet welfare when the sow is loose in the farrowing pen, except for 2-3 days after farrowing:

	0	1	2	3	4	5	6	7	8	9	10	Do not know
With pens of 4 m2 (as per EU norm)	(2) ❑	(3) ❑	(4) ❑	(12) ❑	(13) ❑	(14) ❑	(15) ❑	(16) ❑	(17) ❑	(18) ❑	(19) ❑	(20) ❑
With pens of 6 m2	(2) ❑	(3) ❑	(4) ❑	(12) ❑	(13) ❑	(14) ❑	(15) ❑	(16) ❑	(17) ❑	(18) ❑	(19) ❑	(20) ❑
With pens of 7.5 m2	(2) ❑	(3) ❑	(4) ❑	(12) ❑	(13) ❑	(14) ❑	(15) ❑	(16) ❑	(17) ❑	(18) ❑	(19) ❑	(20) ❑
With pens of 10 m2	(2) ❑	(3) ❑	(4) ❑	(12) ❑	(13) ❑	(14) ❑	(15) ❑	(16) ❑	(17) ❑	(18) ❑	(19) ❑	(20) ❑

Piglet welfare when the sow is loose in the all times:

	0	1	2	3	4	5	6	7	8	9	10	Do not know
With pens of 4 m2 (as per EU norm)	(2) ❑	(3) ❑	(4) ❑	(12) ❑	(13) ❑	(14) ❑	(15) ❑	(16) ❑	(17) ❑	(18) ❑	(19) ❑	(20) ❑
With pens of 6 m2	(2) ❑	(3) ❑	(4) ❑	(12) ❑	(13) ❑	(14) ❑	(15) ❑	(16) ❑	(17) ❑	(18) ❑	(19) ❑	(20) ❑
With pens of 7.5 m2	(2) ❑	(3) ❑	(4) ❑	(12) ❑	(13) ❑	(14) ❑	(15) ❑	(16) ❑	(17) ❑	(18) ❑	(19) ❑	(20) ❑
With pens of 10 m2	(2) ❑	(3) ❑	(4) ❑	(12) ❑	(13) ❑	(14) ❑	(15) ❑	(16) ❑	(17) ❑	(18) ❑	(19) ❑	(20) ❑
With the sow loose in farrow hut with outdoor access	(2) ❑	(3) ❑	(4) ❑	(12) ❑	(13) ❑	(14) ❑	(15) ❑	(16) ❑	(17) ❑	(18) ❑	(19) ❑	(20) ❑

What score would you give for <u>piglet welfare</u> in a production system with the following practice for weaning? The scale is from 0 to 10 where 0 represents minimal welfare for the piglet and 10 represents the best possible welfare for the piglet.

Note: The welfare of the sow in this production system is addressed in another question.

	0	1	2	3	4	5	6	7	8	9	10	Do not know
Piglets are weaned after 14 days (does not meet EU requirements)	(2) ❑	(3) ❑	(4) ❑	(12) ❑	(13) ❑	(14) ❑	(15) ❑	(16) ❑	(17) ❑	(18) ❑	(19) ❑	(20) ❑
Piglets are weaned after 21 days (EU requirement is 28 days but special dispensation to 21 days can be given)	(2) ❑	(3) ❑	(4) ❑	(12) ❑	(13) ❑	(14) ❑	(15) ❑	(16) ❑	(17) ❑	(18) ❑	(19) ❑	(20) ❑
Piglets are weaned after 28 days	(2) ❑	(3) ❑	(4) ❑	(12) ❑	(13) ❑	(14) ❑	(15) ❑	(16) ❑	(17) ❑	(18) ❑	(19) ❑	(20) ❑
Piglets are weaned after 30 days	(2) ❑	(3) ❑	(4) ❑	(12) ❑	(13) ❑	(14) ❑	(15) ❑	(16) ❑	(17) ❑	(18) ❑	(19) ❑	(20) ❑
Piglets are weaned after 40 days	(2) ❑	(3) ❑	(4) ❑	(12) ❑	(13) ❑	(14) ❑	(15) ❑	(16) ❑	(17) ❑	(18) ❑	(19) ❑	(20) ❑
Piglets are weaned after 49 days	(2) ❑	(3) ❑	(4) ❑	(12) ❑	(13) ❑	(14) ❑	(15) ❑	(16) ❑	(17) ❑	(18) ❑	(19) ❑	(20) ❑
Piglets are weaned after 70 days	(2) ❑	(3) ❑	(4) ❑	(12) ❑	(13) ❑	(14) ❑	(15) ❑	(16) ❑	(17) ❑	(18) ❑	(19) ❑	(20) ❑

What score would you give for <u>piglet welfare</u> in a production system with the following practice for tail docking? The scale is from 0 to 10 where 0 represents minimal welfare for the piglet and 10 represents the best possible welfare for the piglet.

	0	1	2	3	4	5	6	7	8	9	10	Do not know
Piglets are tail docked without pain relief or anaesthesia (does not meet EU requirements)	(2) ❑	(3) ❑	(4) ❑	(12) ❑	(13) ❑	(14) ❑	(15) ❑	(16) ❑	(17) ❑	(18) ❑	(19) ❑	(20) ❑
Until day 4, piglets are tail docked without pain relief. After day 4, piglets that are	(2) ❑	(3) ❑	(4) ❑	(12) ❑	(13) ❑	(14) ❑	(15) ❑	(16) ❑	(17) ❑	(18) ❑	(19) ❑	(20) ❑

	0	1	2	3	4	5	6	7	8	9	10	Do not know
tail docked receive general pain relief and full anaesthesia (meets EU requirements)												
Piglets are only tail docked with general pain relief and full anaesthesia	(2) ❑	(3) ❑	(4) ❑	(12) ❑	(13) ❑	(14) ❑	(15) ❑	(16) ❑	(17) ❑	(18) ❑	(19) ❑	(20) ❑
Piglets are not tail docked and there are no requirements to reduce tail biting	(2) ❑	(3) ❑	(4) ❑	(12) ❑	(13) ❑	(14) ❑	(15) ❑	(16) ❑	(17) ❑	(18) ❑	(19) ❑	(20) ❑
Piglets are not tail docked and tail biting is only tolorated to a limited degree	(2) ❑	(3) ❑	(4) ❑	(12) ❑	(13) ❑	(14) ❑	(15) ❑	(16) ❑	(17) ❑	(18) ❑	(19) ❑	(20) ❑

What score would you give for piglet welfare in a production system with the following practice for surgical castration? The scale is from 0 to 10 where 0 represents minimal welfare for the piglet and 10 represents the best possible welfare for the piglet.
Note: Include the effect on the welfare of the piglets relating to both the castration itself and to differences in behaviour in castrated and non-castrated boars

	0	1	2	3	4	5	6	7	8	9	10	Do not know
Piglets are surgically castrated without any anesthesia, analgesia or general pain relief (does not meet EU requirements)	(2) ❑	(3) ❑	(4) ❑	(12) ❑	(13) ❑	(14) ❑	(15) ❑	(16) ❑	(17) ❑	(18) ❑	(19) ❑	(20) ❑
Until day 7, piglets are surgically castrated without any anesthesia, analgesia or general pain relief. After day 7, piglets are castrated with	(2) ❑	(3) ❑	(4) ❑	(12) ❑	(13) ❑	(14) ❑	(15) ❑	(16) ❑	(17) ❑	(18) ❑	(19) ❑	(20) ❑

	0	1	2	3	4	5	6	7	8	9	10	Do not know
general pain relief (does not meet EU requirements)												
Until day 7, piglets are surgically castrated without any anesthesia, analgesia or general pain relief. After day 7, piglets are castrated with local analgesia via an injection and with general pain relief (meets EU requirements)	(2) ❑	(3) ❑	(4) ❑	(12) ❑	(13) ❑	(14) ❑	(15) ❑	(16) ❑	(17) ❑	(18) ❑	(19) ❑	(20) ❑
Until day 7, piglets are surgically castrated without any anaesthesia but with general pain relief. After day 7, piglets are castrated with local analgesia via an injection and with general pain relief	(2) ❑	(3) ❑	(4) ❑	(12) ❑	(13) ❑	(14) ❑	(15) ❑	(16) ❑	(17) ❑	(18) ❑	(19) ❑	(20) ❑
Piglets are only surgically castrated with local analgesia via an injection and with general pain relief	(2) ❑	(3) ❑	(4) ❑	(12) ❑	(13) ❑	(14) ❑	(15) ❑	(16) ❑	(17) ❑	(18) ❑	(19) ❑	(20) ❑
Piglets are only castrated with full anaesthesia through inhalation and with general pain relief	(2) ❑	(3) ❑	(4) ❑	(12) ❑	(13) ❑	(14) ❑	(15) ❑	(16) ❑	(17) ❑	(18) ❑	(19) ❑	(20) ❑
Piglets are not castrated	(2) ❑	(3) ❑	(4) ❑	(12) ❑	(13) ❑	(14) ❑	(15) ❑	(16) ❑	(17) ❑	(18) ❑	(19) ❑	(20) ❑

WEANERS AND FINISHERS

What score would you give for <u>pig welfare</u> in a production system with the following space requirements for smaller pigs of 25-30 kg? The scale is from 0 to 10 where 0 represents minimal welfare for the piglet and 10 represents the best possible welfare for the piglet

regarding this feature.

	**0**	**1**	**2**	**3**	**4**	**5**	**6**	**7**	**8**	**9**	**10**	**Do not know**
0.2 m2 per pig (does not meet EU requirements)	(2) ❑	(3) ❑	(4) ❑	(12) ❑	(13) ❑	(14) ❑	(15) ❑	(16) ❑	(17) ❑	(18) ❑	(19) ❑	(20) ❑
0.3 m2 per pig (meets EU requirements)	(2) ❑	(3) ❑	(4) ❑	(12) ❑	(13) ❑	(14) ❑	(15) ❑	(16) ❑	(17) ❑	(18) ❑	(19) ❑	(20) ❑
0.4 m2 per pig	(2) ❑	(3) ❑	(4) ❑	(12) ❑	(13) ❑	(14) ❑	(15) ❑	(16) ❑	(17) ❑	(18) ❑	(19) ❑	(20) ❑
0.5 m2 per pig	(2) ❑	(3) ❑	(4) ❑	(12) ❑	(13) ❑	(14) ❑	(15) ❑	(16) ❑	(17) ❑	(18) ❑	(19) ❑	(20) ❑
1 m2 per pig	(2) ❑	(3) ❑	(4) ❑	(12) ❑	(13) ❑	(14) ❑	(15) ❑	(16) ❑	(17) ❑	(18) ❑	(19) ❑	(20) ❑
1 m2 per pig, with outdoor access	(2) ❑	(3) ❑	(4) ❑	(12) ❑	(13) ❑	(14) ❑	(15) ❑	(16) ❑	(17) ❑	(18) ❑	(19) ❑	(20) ❑

What score would you give for <u>pig welfare</u> in a production system with the following space requirements for finisher pigs of 100-110 kg? The scale is from 0 to 10 where 0 represents minimal welfare for the piglet and 10 represents the best possible welfare for the piglet.

	**0**	**1**	**2**	**3**	**4**	**5**	**6**	**7**	**8**	**9**	**10**	**Do not know**
0.5 m2 per pig (does not meet EU requirements)	(2) ❑	(3) ❑	(4) ❑	(12) ❑	(13) ❑	(14) ❑	(15) ❑	(16) ❑	(17) ❑	(18) ❑	(19) ❑	(20) ❑
0.65 m2 per pig (meets EU requirements)	(2) ❑	(3) ❑	(4) ❑	(12) ❑	(13) ❑	(14) ❑	(15) ❑	(16) ❑	(17) ❑	(18) ❑	(19) ❑	(20) ❑
0.85 m2 per pig	(2) ❑	(3) ❑	(4) ❑	(12) ❑	(13) ❑	(14) ❑	(15) ❑	(16) ❑	(17) ❑	(18) ❑	(19) ❑	(20) ❑
1 m2 per pig	(2) ❑	(3) ❑	(4) ❑	(12) ❑	(13) ❑	(14) ❑	(15) ❑	(16) ❑	(17) ❑	(18) ❑	(19) ❑	(20) ❑
1.3 m2 per pig	(2) ❑	(3) ❑	(4) ❑	(12) ❑	(13) ❑	(14) ❑	(15) ❑	(16) ❑	(17) ❑	(18) ❑	(19) ❑	(20) ❑
2 m2 per pig	(2) ❑	(3) ❑	(4) ❑	(12) ❑	(13) ❑	(14) ❑	(15) ❑	(16) ❑	(17) ❑	(18) ❑	(19) ❑	(20) ❑

	0	1	2	3	4	5	6	7	8	9	10	Do not know
2 m2 per pig, with outdoor access	(2) ❑	(3) ❑	(4) ❑	(12) ❑	(13) ❑	(14) ❑	(15) ❑	(16) ❑	(17) ❑	(18) ❑	(19) ❑	(20) ❑

What score would you give for <u>pig welfare</u> (100-110 kg) in a production system with the following restrictions for slatted floors? The scale is from 0 to 10 where 0 represents minimal welfare for the piglet and 10 represents the best possible welfare for the piglet.

	0	1	2	3	4	5	6	7	8	9	10	Do not know
Fully slatted floors (meets EU requirements)	(2) ❑	(3) ❑	(4) ❑	(12) ❑	(13) ❑	(14) ❑	(15) ❑	(16) ❑	(17) ❑	(18) ❑	(19) ❑	(20) ❑
Slatted floors, max. two thirds of total floor area	(2) ❑	(3) ❑	(4) ❑	(12) ❑	(13) ❑	(14) ❑	(15) ❑	(16) ❑	(17) ❑	(18) ❑	(19) ❑	(20) ❑
Slatted floors, max. half of total floor area	(2) ❑	(3) ❑	(4) ❑	(12) ❑	(13) ❑	(14) ❑	(15) ❑	(16) ❑	(17) ❑	(18) ❑	(19) ❑	(20) ❑
Slatted floors, max. one third of total floor area	(2) ❑	(3) ❑	(4) ❑	(12) ❑	(13) ❑	(14) ❑	(15) ❑	(16) ❑	(17) ❑	(18) ❑	(19) ❑	(20) ❑
No slatted floors	(2) ❑	(3) ❑	(4) ❑	(12) ❑	(13) ❑	(14) ❑	(15) ❑	(16) ❑	(17) ❑	(18) ❑	(19) ❑	(20) ❑

GENERAL ISSUES

What score would you give for pig welfare in a production system with the following practice for bedding? The scale is from 0 to 10 where 0 represents minimal welfare for the piglet and 10 represents the best possible welfare for the piglet regarding this feature.

	0	1	2	3	4	5	6	7	8	9	10	Do not know
No bedding (does not meet EU requirements)	(2) ❑	(3) ❑	(4) ❑	(12) ❑	(13) ❑	(14) ❑	(15) ❑	(16) ❑	(17) ❑	(18) ❑	(19) ❑	(20) ❑

	0	1	2	3	4	5	6	7	8	9	10	Do not know
Bedding to be picked from a rack (meets EU requirements)	(2) ❑	(3) ❑	(4) ❑	(12) ❑	(13) ❑	(14) ❑	(15) ❑	(16) ❑	(17) ❑	(18) ❑	(19) ❑	(20) ❑
Bedding on the floor	(2) ❑	(3) ❑	(4) ❑	(12) ❑	(13) ❑	(14) ❑	(15) ❑	(16) ❑	(17) ❑	(18) ❑	(19) ❑	(20) ❑
Plenty of permanent bedding on the floor	(2) ❑	(3) ❑	(4) ❑	(12) ❑	(13) ❑	(14) ❑	(15) ❑	(16) ❑	(17) ❑	(18) ❑	(19) ❑	(20) ❑
Plenty of permanent bedding on the floor and in the lying area	(2) ❑	(3) ❑	(4) ❑	(12) ❑	(13) ❑	(14) ❑	(15) ❑	(16) ❑	(17) ❑	(18) ❑	(19) ❑	(20) ❑

What score would you give for pig welfare in a production system with the following practice for investigation and manipulation material? The scale is from 0 to 10 where 0 represents minimal welfare for the piglet and 10 represents the best possible welfare for the piglet.

	0	1	2	3	4	5	6	7	8	9	10	Do not know
No material on the floor (does not meet EU requirements)	(2) ❑	(3) ❑	(4) ❑	(12) ❑	(13) ❑	(14) ❑	(15) ❑	(16) ❑	(17) ❑	(18) ❑	(19) ❑	(20) ❑
Manipulation material includes a chain or car tyres (meets EU requirements)	(2) ❑	(3) ❑	(4) ❑	(12) ❑	(13) ❑	(14) ❑	(15) ❑	(16) ❑	(17) ❑	(18) ❑	(19) ❑	(20) ❑
Manipulation material includes a piece of wood	(2) ❑	(3) ❑	(4) ❑	(12) ❑	(13) ❑	(14) ❑	(15) ❑	(16) ❑	(17) ❑	(18) ❑	(19) ❑	(20) ❑
Manipulation material includes a piece of wood or saw dust	(2) ❑	(3) ❑	(4) ❑	(12) ❑	(13) ❑	(14) ❑	(15) ❑	(16) ❑	(17) ❑	(18) ❑	(19) ❑	(20) ❑
Manipulation material includes straw or sphagnum	(2) ❑	(3) ❑	(4) ❑	(12) ❑	(13) ❑	(14) ❑	(15) ❑	(16) ❑	(17) ❑	(18) ❑	(19) ❑	(20) ❑

What score would you give for pig welfare in a production system with the following practice for keeping animals in pen groups? The scale is from 0 to 10 where 0 represents minimal welfare for the piglet and 10 represents the best possible welfare for the piglet.

	0	1	2	3	4	5	6	7	8	9	10	Do not know
Pigs are mixed in new groups several times during their life (meets EU requirements)	(2) ❑	(3) ❑	(4) ❑	(12) ❑	(13) ❑	(14) ❑	(15) ❑	(16) ❑	(17) ❑	(18) ❑	(19) ❑	(20) ❑
Pigs are kept in pen groups for the entire production	(2) ❑	(3) ❑	(4) ❑	(12) ❑	(13) ❑	(14) ❑	(15) ❑	(16) ❑	(17) ❑	(18) ❑	(19) ❑	(20) ❑

What score would you give for pig welfare in a production system with the following practice for transport to slaughter? The scale is from 0 to 10 where 0 represents minimal welfare for the piglet and 10 represents the best possible welfare for the piglet.

	0	1	2	3	4	5	6	7	8	9	10	Do not know
The pigs are transported for 24 hours without a break. After 24 hours, the pigs have a break of 24 hours with food and water, and are transported for a further 24 hours (meets EU requirements)	(2) ❑	(3) ❑	(4) ❑	(12) ❑	(13) ❑	(14) ❑	(15) ❑	(16) ❑	(17) ❑	(18) ❑	(19) ❑	(20) ❑
The pigs are transported for 24 hours without a break	(2) ❑	(3) ❑	(4) ❑	(12) ❑	(13) ❑	(14) ❑	(15) ❑	(16) ❑	(17) ❑	(18) ❑	(19) ❑	(20) ❑
The pigs are transported for 8 hours plus time in the waiting area	(2) ❑	(3) ❑	(4) ❑	(12) ❑	(13) ❑	(14) ❑	(15) ❑	(16) ❑	(17) ❑	(18) ❑	(19) ❑	(20) ❑

	0	1	2	3	4	5	6	7	8	9	10	Do not know
The pigs are transported for 8 hours including time in the waiting area	(2) ❑	(3) ❑	(4) ❑	(12) ❑	(13) ❑	(14) ❑	(15) ❑	(16) ❑	(17) ❑	(18) ❑	(19) ❑	(20) ❑
The pigs are transported for 4 hours plus time in the waiting area	(2) ❑	(3) ❑	(4) ❑	(12) ❑	(13) ❑	(14) ❑	(15) ❑	(16) ❑	(17) ❑	(18) ❑	(19) ❑	(20) ❑
The pigs are transported for 4 hours including time in the waiting area	(2) ❑	(3) ❑	(4) ❑	(12) ❑	(13) ❑	(14) ❑	(15) ❑	(16) ❑	(17) ❑	(18) ❑	(19) ❑	(20) ❑
The pigs are not transported (mobile slaughtering units are used)	(2) ❑	(3) ❑	(4) ❑	(12) ❑	(13) ❑	(14) ❑	(15) ❑	(16) ❑	(17) ❑	(18) ❑	(19) ❑	(20) ❑

EVALUATION OF THE IMPORTANCE OF INDIVIDUAL ANIMAL WELFARE ASPECTS
Finally, we ask you to evaluate the importance of each of the above-mentioned aspects of the production system against other changes in the production that might affect animal welfare.

The scale is from 1 (not important) to 5 (very important). If, for example, you evaluate two different aspects as "very important", they will have the same weight when welfare levels in individual production systems are measured together.

	1	2	3	4	5	Do not know
1. How important is loose-housing for sow welfare?	(3) ❑	(4) ❑	(12) ❑	(13) ❑	(14) ❑	(15) ❑
2. How important is space in the farrowing unit for sow welfare?	(3) ❑	(4) ❑	(12) ❑	(13) ❑	(14) ❑	(15) ❑
3. How important is space for sow welfare when she is loose in group housing (in mating and gestation units)?	(3) ❑	(4) ❑	(12) ❑	(13) ❑	(14) ❑	(15) ❑

	1	2	3	4	5	Do not know
4. How important is nest-building for sow welfare?	(3) ❑	(4) ❑	(12) ❑	(13) ❑	(14) ❑	(15) ❑
5. How important is the weaning age for sow welfare?	(3) ❑	(4) ❑	(12) ❑	(13) ❑	(14) ❑	(15) ❑

	1	2	3	4	5	Do not know
6. How important is space in the farrowing unit for piglet welfare?	(3) ❑	(4) ❑	(12) ❑	(13) ❑	(14) ❑	(15) ❑
7. How important is it for piglet welfare that the sow is loose in the farrowing unit?	(3) ❑	(4) ❑	(12) ❑	(13) ❑	(14) ❑	(15) ❑
8. How important is weaning age for piglet welfare?	(3) ❑	(4) ❑	(12) ❑	(13) ❑	(14) ❑	(15) ❑
9. How important are initiatives regarding tail docking for piglet welfare when there is a risk of tail biting if tails are not docked?	(3) ❑	(4) ❑	(12) ❑	(13) ❑	(14) ❑	(15) ❑
10. How important are initiatives regarding tail docking for piglet welfare when there is no risk of tail biting if tails are not docked?	(3) ❑	(4) ❑	(12) ❑	(13) ❑	(14) ❑	(15) ❑
11. How important are initiatives regarding castration for piglet welfare? Note: Include piglet welfare relating to both the castration itself	(3) ❑	(4) ❑	(12) ❑	(13) ❑	(14) ❑	(15) ❑

	1	2	3	4	5	Do not know
and to differences in behaviour in castrated and non-castrated boars.						

	1	2	3	4	5	Do not know
12. How important is space for weaner welfare?	(3) ❑	(4) ❑	(12) ❑	(13) ❑	(14) ❑	(15) ❑
13. How important is space for finisher welfare?	(3) ❑	(4) ❑	(12) ❑	(13) ❑	(14) ❑	(15) ❑
14. How important are initiatives to limit slatted flooring for weaner welfare?	(3) ❑	(4) ❑	(12) ❑	(13) ❑	(14) ❑	(15) ❑
15. How important are initiatives to limit slatted flooring for finisher welfare?	(3) ❑	(4) ❑	(12) ❑	(13) ❑	(14) ❑	(15) ❑

	1	2	3	4	5	Do not know
16. How important is bedding for pig welfare?	(3) ❑	(4) ❑	(12) ❑	(13) ❑	(14) ❑	(15) ❑
17. How important is access to investigation and manipulation material for pig welfare?	(3) ❑	(4) ❑	(12) ❑	(13) ❑	(14) ❑	(15) ❑
18. How important are pen groups for pig welfare?	(3) ❑	(4) ❑	(12) ❑	(13) ❑	(14) ❑	(15) ❑
19. How important is transportation time for pig welfare?	(3) ❑	(4) ❑	(12) ❑	(13) ❑	(14) ❑	(15) ❑

Thank you for participating in this survey. If you have any comments please list them here.

____

In order to be able to send you an Amazon voucher of 50 Euro, we ask you to write you email below. We will delete this information afterwards.

We thank you for participating in our survey!
